# Functional and Endoscopic Indicators for Percutaneous Endoscopic Gastrostomy (PEG) in Amyotrophic Lateral Sclerosis Patients

**DOI:** 10.3390/jcm7100352

**Published:** 2018-10-14

**Authors:** Bebiana Conde, Natália Martins, Inês Rodrigues, Ana Cláudia Pimenta, João Carlos Winck

**Affiliations:** 1 Pulmonology Department, Centro Hospitalar Trás-os-Montes e Alto Douro, Avenida da Noruega, Vila Real 5000-508, Portugal; ines.rsr@gmail.com (I.R.); anacscpimenta@gmail.com (A.C.P.); 2 Faculty of Medicine, University of Porto, Alameda Prof. Hernâni Monteiro, Porto 4200-319, Portugal; ncmartins@med.up.pt (N.M.); jcwinck@mail.telepac.pt (J.C.W.); 3 Institute for Research and Innovation in Health (i3S), University of Porto, Rua Alfredo Allen, Porto 4200-135, Portugal

**Keywords:** percutaneous endoscopic gastrostomy, Amyotrophic Lateral Sclerosis, functional parameters

## Abstract

(1) Background: Amyotrophic lateral sclerosis (ALS) is a progressive neurodegenerative condition, whose bulbar involvement compromises language, swallowing, and airway protection. When oral nutrition is no longer adequate, percutaneous endoscopic gastroscopy (PEG) may be indicated. However, as exact timing is still debatable, we tried to find it. (2) Methods: A prospective cohort study was performed using fiber-optic endoscopic evaluation of swallowing (FEES), functional evaluation scales (ALS Functional Rating Scale-Revised (ALSFRS-R) and bulbar sub-score (ALSFRS-R-B)), lung function tests (like Forced Vital Capacity (FVC), Cough Peak Flow (CPF)) and anthropometric data. (3) Results: Twenty-three patients were enrolled (mean 65.4 ± 9.1 years, 60.9% males), 12 with spinal-onset. During the study period, 58 FEES were performed (1–4/patients). Even before formal PEG indication, suggestions were given to correct the alterations found. PEG was placed in 12 patients, on average 21.8 months after diagnosis (FVC = 69.9% ± 26.7%, ALSFRS-R-B = 7.7 ± 3.7, ALSFRS-R = 28.9 ± 12.3), and being 91.7% under ventilatory support. ALSFRS-R-B, CPF, FVC, and ALSFRS-R showed significant discriminant ability for PEG placement. Sensitivity and specificity were, respectively, ALSFRS-R-B ≤ 8 (100/90.9), CPF ≤ 205 (83.3), FVC ≤ 74 (83.3/74.2), and ALSFRS-R < 29 (83.3/65.1). (4) Conclusions: FEES provide additional information beyond formal PEG indication. ALSFRS-R-B score ≤ 8 was found as a best functional and noninvasive indicator for PEG placement in ALS patients.

## 1. Introduction

Amyotrophic Lateral Sclerosis (ALS) is a progressive and fatal degenerative neurologic disease, characterized by upper and lower motor neuron dysfunction, causing weakness of oropharyngeal and respiratory muscles, that results in dysphagia and respiratory failure [1]. Bulbar involvement may take place at the onset or throughout its evolution, leading to the development of distinct clinical symptoms. One of the most significant symptoms is dysphagia, as it may favor malnutrition, dehydration and weight loss, as well as aspiration pneumonia. Together, these complications markedly influence mortality rates, which may justify the worse prognosis of bulbar-onset ALS, compared to limb onset ALS [1,2], as both nutritional deficits and malnutrition status are recognized to be associated with mortality in these patients [3]. Thus, nutritional supplementation is very important in the course of disease, and dysphagia management, with a gastrostomy tube, is conceived as one of the cornerstones of symptomatic treatment in ALS patients [1,3,4].

A gastrostomy tube (G tube) can be accomplished by different methods, like percutaneous endoscopic gastrostomy (PEG), radiological gastrostomy (RIG) or per-oral image-guided gastrostomy (PIG), that are similar in efficacy [1,5], although some authors have reported a better survival in ALS patients with moderate to severe respiratory impairment under RIG than PEG [6,7,8].

The optimal timing to perform PEG is controversial; however, the American Academy of Neurological Societies and European Federation of Neurological Guidelines recommend G tube placement preferably when Forced Vital Capacity (FVC) > 50%, since it reduces procedure risk, besides being associated with a better prognosis in ALS patients [1,9]. Nevertheless, before placing G tube, the presence of dysphagia in ALS patients should be ascertained, in order to assess the need for food consistency adjustment or body positioning during swallowing. There are some scales used to classify dysphagia and the gold standard to the study is a video fluoroscopy and video endoscopy of swallowing [10,11,12].

Some functional indicators have been examined in ALS patients to study dysphagia and even to perform PEG, like FVC, Voluntary Cough Airflow, ALS Functional Rating Scale-Revised (ALSFRS-R) [12,13,14], and lastly a risk stratification questionnaire for PEG placement [15]. This subject has been a “hot topic”; however, none of the previous investigations were able to identify the optimal indicators to place G tube, which is considered a safe procedure, even in the presence of severe respiratory impairment [1,8].

Accordingly, we performed a longitudinal prospective study using fiber-optic endoscopic evaluation of swallowing (FEES), simultaneously to respiratory function, and functional evaluation scales (ALSFRS-R and bulbar sub-score (ALSFRS-R-B)) assessment, trying to find the best functional or endoscopic indicator to PEG placement.

## 2. Experimental Section

### 2.1. Participants

A homogeneous sample of twenty-three Caucasian individuals with probable or definitive (Revised El-Escorial Criteria) ALS diagnosis were enrolled and followed-up quarterly, until performing PEG, patient refusal, or death, between October 2016 and March 2018. This study had the approval of local Ethics Committee (Centro-Hospitalar Trás-os-Montes e Alto Douro, Vila Real, Portugal) and informed consent was obtained from all patients.

### 2.2. Procedures

Every 3 months, participants performed respiratory functional evaluation, with Cough Peak Flow (CPF) being measured using Mini Wright Peak Flow Meter, as described by Winck et al. [16] and Suárez et al. [17]. Peak Expiratory Flow (PEF) was also measured using Mini Wright Peak Flow Meter and PEF/CPF was calculated. FVC (seated and supine) was measured using MicroLab™ Spirometer (CareFusion, CareFusion, Parsippany, NJ, USA) and Maximal Inspiratory Pressure (PImax) and Maximal Expiratory Pressure (PEmax) were determined using MicroRPM™ (CareFusion, Mettawa, IL, USA). These values were determined according to ATS/ERS guidelines [18,19]. ALSFRS-R form was completed, and patients were subjected to FEES according to the published protocol by Castro et al. [20], using the bronchoscope Pentax^®^ EB-1570K (channel diameter 2.0 mm and distal tip diameter 5.5 mm) (Tokyo, Japan), and video recording. Bronchoscope distal extremity was placed in the transition between oropharyngeal and nasopharyngeal, prior to the epiglottis, with nasal introduction, without local anesthesia or sedation, only with lidocaine gel-bronchoscope lubrification. 

Four different types of food consistency were used: (A) 100 mL of water with 2 mL methylene blue; (B) 100 mL of water with 2 mL methylene blue and 6 g of thickener (Nutilis^®^ (produced by Milupa, Friedrichsdorf, Germany)); (C) 100 mL of water with 2 mL methylene blue and 10 g of thickener (Nutilis^®^); (D) solid cake with 2 mL methylene blue. A and B consistence was given with 5 mL, 10 mL, and 15 mL spoons, while C and D, with 2 spoons of 15 mL.

The presence of laryngeal penetration or tracheal-laryngeal aspiration of food, non-correctable with thickener, was an indication to PEG placement.

### 2.3. Statistical Analyses

Categorical variables were described as absolute values (N) and relative frequencies, and continuous variables as mean and standard deviation (SD). Two sample *t*-test were used to compare the continuous characteristics among subgroups. Sensitivity, specificity, and receiver operator characteristics (ROC) were determined to assess the discriminative ability of the different functional and endoscopic indicators (ALSFRS-R, ALSFRS-R-B, CPF and FVC) on PEG placement. All data were analyzed using Statistical Package for the Social Sciences (SPSS (IBM Corp., Inc., Armonk, NY, USA)) software, version 25.0, with alpha set at 0.05.

## 3. Results

### 3.1. Patients’ Characteristics and Functional Parameters

From the twenty-three patients included, with a mean age of 65.4 ± 9.1 years, 14 (60.9%) were male and 12 (52.2%) had spinal onset, all of them under specific therapy with riluzole. At the time of diagnosis, 13 patients had PEF/CPF > 1 (56.5%) and mean FVC of 84.6% ± 27.4% (Table 1).

**Figure 1:**
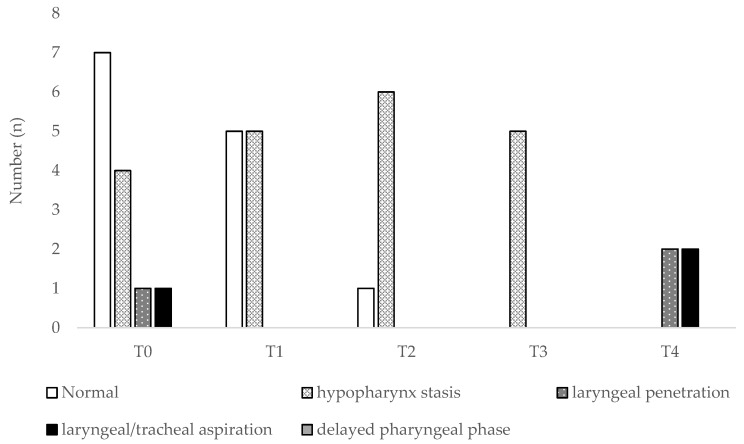
Fiber-optic endoscopic evaluation of swallowing (FEES) findings in Amyotrophic lateral sclerosis (ALS) spinal-onset patients. T0 stands for the evaluation at the time of diagnosis; T1, T2, T3 and T4 stand for follow-up trimesters.

**Figure 2:**
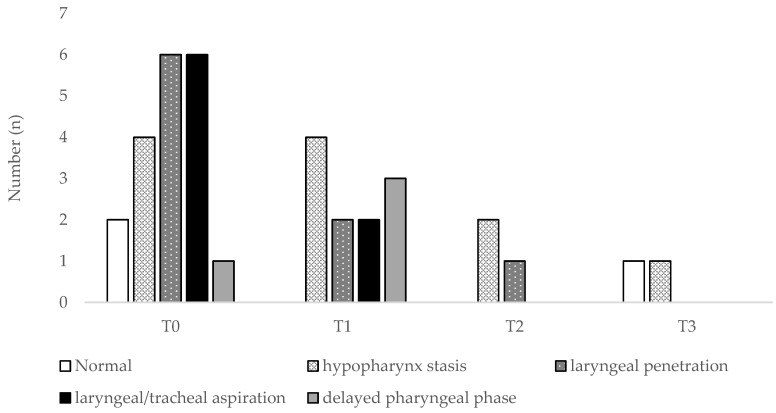
FEES findings in ALS bulbar-onset patients. T0 stands for the evaluation at the time of diagnosis, T1, T2 and T3 stand for follow-up trimesters.

In the study period, 58 FEES were performed (1–5 per patient), as shown in Figure 1 and Figure 2. Interestingly, patients often showed various simultaneous swallowing abnormalities. Two patients (one with ALS-B and one with ALS-S) even appeared to have fluctuations between stasis findings and normal swallowing, rather than continuous progression. Even before formal PEG indication, we were able to propose food consistency alterations, such as food consistency increase (*n* = 10) or decrease (*n* = 1). Fifteen of these patients (65.2%) engaged the follow-up protocol. We performed PEG in 12 of them, 1 patient dropped our study and 2 died prior to PEG, due to rapid disease progression, triggering cardiorespiratory failure. PEG was performed on average 21.8 months after diagnosis, without complications or 30 days-mortality. At 3 months, only a case of pneumonia was reported, but no new events were stated at 6 months. After PEG placement, 4 patients complained of local pain (with an average value of 4 in 10, according to the Visual Analogic Score-VAS), which was controlled with paracetamol 3–4 g/day for 24–48 h.

Mean FVC was 69.9% ± 26.7%, CPF 255.6 ± 176.7 L/min, ALSFRS-R 28.9 ± 12.3 and ALSFRS-R bulbar score 7.7 ± 3.7. Among patients where PEG was done, 9 (75%) had bulbar onset. Almost all the patients (91.7%) in whom PEG was performed were already under ventilatory support, none with tracheostomy (starting non-invasive mechanical ventilation 17.6 months after diagnosis, and accomplishing on average 11.3 ± 4.4 h/day). Only four of our patients had a significant weight loss before PEG placement (6.5% ± 1.8%), and these subjects continued to show sustained weight loss after the procedure. Before PEG placement, we reported respiratory events that led to hospitalization in five patients. This number raised to eight patients after the procedure, mainly justified by disease progression.

### 3.2. Discriminant Ability of Functional or Endoscopic Indicators for Predicting PEG

In this sample, four clinical and functional indicators demonstrated significant ability to be considered PEG indicators, compared to FEES indication. Gender-based significant differences were found in ALSFRS-R (34.4 ± 9.4 for males and 20.3 ± 11.7 for females, *p* = 0.005), ALSFRS-R-B (9.5 ± 3.0 for males and 4.9 ± 2.8 for females, *p* = 0.001) and FEES (specifically at time T6, *p* = 0.007) assessments. These differences may be justified by the predominance of females in the bulbar phenotype (Table 1).

**Figure 3:**
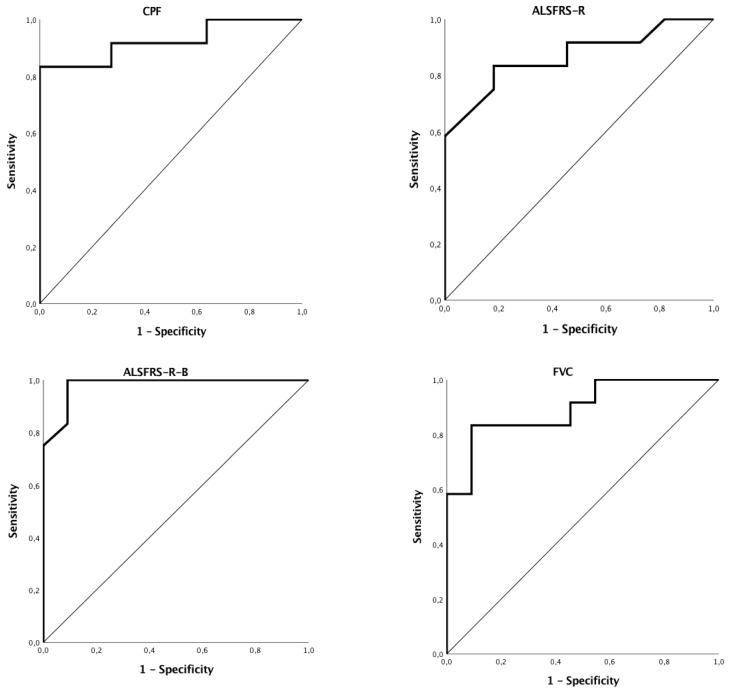
Receiver operator curves (ROC) for functional and endoscopic measures that were considered significant to percutaneous endoscopic gastrostomy (PEG) indication. ALSFRS-R, ALS Functional Rating Scale-Revised; ALSFRS-R-B, ALS Functional Rating Scale-Revised bulbar sub-score; CPF, cough peak flow; FVC, forced vital capacity.

The respective areas under the curve (AUC) and *p* values were as follows: ALSFRS-R-B (AUC = 0.98; *p* < 0.001), CPF (AUC = 0.92; *p* = 0.001), FVC (AUC = 0.89; *p* = 0.001), and ALSFRS-R (AUC = 0.87; *p* = 0.003). Figure 3 displays ROC obtained graphs for ALSFRS-R-B, CPF, FVC and ALSFRS-R. Sensitivity (S) values were of 100%, 83.3%, 83.3%, and 83.3%, respectively, for ALSFRS-R-B, CPF, FVC and ALSFRS-R. Specificity (E) values were of 90.9%, 83.3%, 74.2% and 65.1%, respectively, for ALSFRS-B, CPF, FVC and ALSFRS-R. The cut-offs obtained for these measures were, respectively, ALSFRS-R-B ≤ 8, CPF ≤ 205, FVC ≤ 74 and ALSFRS-R < 29.

### 3.3. Survival

Of all 23 patients enrolled, ALS bulbar onset phenotype exhibited the shortest median survival, 31 months, while ALS spinal had the longest, 52 months. The ALS bulbar onset survival was significantly different from the ALS spinal onset (*p* = 0.014). 

During the follow-up period, 6 patients died after PEG placement (5 of respiratory failure and 1 of cardiorespiratory arrest). The first patient died 3 months after the procedure due to respiratory failure, which was preceded by pneumonia (survival time—22 months). The other four patients who died by respiratory failure, died respectively 4 months (survival time—36 months), 6 months (survival time—36 months), 7 months (survival time—15 months) and 12 months (total survival time—109 months) after PEG placement. Finally, the patient who suffered cardiorespiratory arrest died 13 months after PEG placement (survival time—25 months). We highlight that non-invasively ventilated patients have decided in advance and consciously.

## 4. Discussion

In our cohort, FEES was a helpful tool guiding adjustment of food consistency, even before PEG placement indications, as recommended by Burgos et al. [4]. As previously reported, there is a strong correlation between disease severity (evaluated by ALSFRS-R and ALSFRS-R-B) and dysphagia severity, assessed by pre and post swallowing residues [21]. FEES is well established as providing information about bolus aspiration. Beyond that, it also shows progressive swallowing abnormalities related to disease severity [21]. Despite the small sample size, our study suggests that the use of a clinical and functional indicator for PEG placement in ALS patients can be a valuable tool.

In fact, we found ALSFRS-R-B, CPF, FVC and ALSFRS-R as reliable indicators for PEG placement, being highly sensible and specific. ALSFRS-R-B ≤ 8 showed a remarkably high S and E (100% and 90.9%, respectively), suggesting this cut-off value as the best noninvasive indicator for PEG placement described so far. The other functional indicators described also add significant predictive values, although not as high. CPF ≤ 205 demonstrated an S and E of 83.3%; FVC ≤ 74 showed a S of 83.3% and E of 74.2%; and ALSFRS-R < 29 had a S of 83.3% with 65.1% of E.

So far, the literature has suggested that PEG placement should be guided by the presence of symptoms related to swallowing disorders, such as dysphagia or asthenia during eating, weight loss or respiratory infections, due to food content aspiration, especially in the presence of relatively preserved respiratory function (FVC > 50%), given the high risk of procedure’ complications in the presence of severe functional impairment [9,22].

As the main limitations of our study, beyond the low size of the cohort, we would like to highlight that the FEES protocol remains qualitative, and also that the bronchoscope used was longer and had a higher caliber compared to the laryngoscope. On the other hand, this aspect can also be considered an advantage, as it favored food aspiration from the larynx and trachea in two patients.

To date, no precise indicators have been established to optimize PEG placement time, although some scales and functional parameters have already been tested [23]. In line with what has been established as indicators for the onset of ventilatory support, namely the presence of inherent symptoms to hypoventilation and functional impairment with FVC < 70% and PImax < 60 cmH_2_O, a CPF < 300 L/min was also suggested as a good indicator of the need for short-term ventilatory support, and CPF < 270 L/min as indicator for cough assistance [9,24]. In this study, we now present a functional and clinical indicator for PEG placement, with 100% S if ALSFRS-R-B ≤ 8 and 100% E if ALSFRS-R-B ≤ 6, in the same line as already described by Rooney et al. [13], regarding its discriminatory power in spinal and bulbar phenotypes. 

Our findings, if replicated in other series, hopefully with larger cohorts, may suggest that our approach could replace the current gold standards (swallowing videoendoscopy or/and videofluoroscopy) [22], in PEG placement decision.

## 5. Conclusions

Endoscopic swallowing evaluation provides additional information beyond the formal PEG indication. ALSFRS-R bulbar score ≤ 8 has good sensitivity and specificity, and thus may be considered the best functional and noninvasive indicator for PEG placement in ALS patients.

## Figures and Tables

**Table 1:** Patient’s demographic data and respiratory function evaluation at diagnosis.

Phenotypes	ALS Spinal Onset	ALS Bulbar Onset	ALS Total
Age (years)	61.3 ± 8.1	69.7 ± 8.3	65.4 ± 9.1
Gender			
Males	10 (83.3%)	4 (36.4%)	14 (60.9%)
Females	2 (16.7%)	7 (63.6%)	9 (39.1%)
FVC (%)	91.1 ± 18.6	77.4 ± 34.0	84.6 ± 27.4
PEmax (cm H_2_O)	88.4 ± 34.5	56.0 ± 45.6	72.9 ± 42.6
PImax (cm H_2_O)	78.0 ± 20.3	34.7 ± 23.5	57.3 ± 30.8
CPF (L/min)	319.6 ± 70.6	205.4 ± 147.1	265.0 ± 125.4
PEF/CPF			
<1	4 (33.3%)	3 (27.3%)	7 (30.4%)
0	0	1 (9.1%)	1 (4.3%)
≥1	8 (66.7%)	7 (63.6%)	15 (65.3%)

ALS, Amyotrophic Lateral Sclerosis; CPF, Cough Peak Flow; FVC, Forced Vital Capacity; PEF, Peak Expiratory Flow; PImax, Maximal Inspiratory Pressure; PEmax, Maximal Expiratory Pressure.
